# Simultaneous evaluation of diagnostic marker utility for enzootic bovine leukosis

**DOI:** 10.1186/s12917-019-2158-4

**Published:** 2019-11-09

**Authors:** Misako Konishi, Sota Kobayashi, Taeko Tokunaga, Yuzumi Chiba, Toshiyuki Tsutsui, Shozo Arai, Ken-ichiro Kameyama, Takehisa Yamamoto

**Affiliations:** 1 Epidemiology Unit, Division of Viral Disease and Epidemiology, National Institute of Animal Health, NARO 3-1-5 Kannondai, Tsukuba, Ibaraki 305-0856 Japan; 2 Parasitic Disease Unit, Division of Bacterial and Parasitic Disease, National Institute of Animal Health, NARO 3-1-5 Kannondai, Tsukuba, Ibaraki 305-0856 Japan; 3 Kumamoto Meat Inspection Office, 1341, Shichijomachisosaki, Kikuchi, Kumamoto, Kumamoto 861-1344 Japan; 4 Iwate Prefecture Central Livestock Hygiene Service Center, 390-5 Sunagome, Takizawa, Iwate 020-0605 Japan; 5 Director-General, National Institute of Animal Health, NARO 3-1-5 Kannondai, Tsukuba, Ibaraki 305-0856 Japan; 6 Clinical Biochemistry Unit, Division of Pathology and Pathophysiology, National Institute of Animal Health, NARO 3-1-5 Kannondai, Tsukuba, Ibaraki, 305-0856 Japan; 7 National Institute of Animal Health, NARO, Exotic Diseases Research Station Josuihoncho, Kodaira, Tokyo 187-0022 Japan

**Keywords:** Enzootic bovine leukosis, Biomarker, Cattle, Thymidine kinase, Lactate dehydrogenase

## Abstract

**Background:**

Enzootic bovine leukosis (EBL) is a disease of cattle caused by bovine leukemia virus (BLV). More than 60% of BLV-infected cattle remain subclinical and are thus referred to as aleukemic (AL) cattle. Approximately 30% of infected cattle show a relatively stable increase in the number of B lymphocytes; these cattle are termed persistent lymphocytosis (PL) cattle. A small percentage of infected cattle develop BLV-induced B cell lymphoma (EBL) and are called EBL cattle. Due to the increase in the number of BLV-infected cattle, the number of EBL cattle has featured a corresponding increase over recent years in Japan. Several diagnostic criteria for EBL (e.g., enlarged superficial lymph nodes, protrusion of the eye, increased peripheral blood lymphocyte, etc.) are used for on-farm diagnosis and antemortem tests at slaughterhouses. Since the slaughter of EBL cattle for human consumption is not allowed, on-farm detection of EBL cattle is important for reducing the economic loss incurred by farms. Therefore, establishing new diagnostic markers to improve the efficiency and accuracy of the antemortem detection of EBL cattle is a critical, unmet need. To simultaneously evaluate the utility of candidate markers, this study measured the values of each marker using the blood samples of 687 cattle with various clinical statuses of BLV infection (EBL, PL, AL and non-infected cattle).

**Results:**

Sensitivity (Se) and specificity (Sp) were highest for the serum thymidine kinase (TK) followed by the serum lactate dehydrogenase (LDH) isozyme 2. The number of peripheral blood lymphocytes and proviral load in peripheral blood had the lowest Se and Sp. The values of all markers other than TK were influenced by the sex of the tested cattle.

**Conclusions:**

Although tLDH and its isozymes (LDHs) may be influenced by the sex of the tested cattle, the high accuracy of TK and LDH2 as well as accessibility and simplicity of the protocol used to measure these enzymes recommend the utility of TK and LDHs for EBL cattle detection. Using these markers for screening followed by the application of existing diagnostic criteria may improve the efficiency and accuracy of EBL cattle detection on farms, thereby contributing to the reduction of economic losses in farms.

## Background

Enzootic bovine leukosis (EBL) is a disease of cattle caused by bovine leukemia virus (BLV), which belongs to *Deltaretrovirus* in the family *Retroviride*. BLV infection causes various clinical states. The majority (> 60%) of BLV-infected cattle remain subclinical; these cattle are termed aleukemic (AL) cattle. Approximately 30% of infected cattle exhibit a relatively stable increase in the number of B lymphocytes; these cattle are termed persistent lymphocytosis (PL) cattle. Only a small percentage of BLV-infected cattle develop BLV-induced B cell lymphoma (EBL). Despite the low incidence of EBL among BLV-infected cattle, the number of EBL cattle has been increasing in Japan, and more than 2000 EBL cattle have been detected annually over recent years. Since the carcasses of EBL cattle cannot be slaughtered for human consumption, they are fully disposed at slaughterhouses in Japan [1]. Moreover, since the viral load in the blood of EBL cattle is higher than those in AL and PL cattle, EBL cattle in farms have a higher risk of spreading BLV through farms. To mitigate economic loss and prevent disease spread, the early detection and culling of EBL cattle is critical. In Japan, the following manifestations are often used as diagnostic criteria of EBL for on-farm diagnosis: emaciation, enlarged superficial lymph nodes, protrusion of the eye, palpable tumor mass in body cavity, increased peripheral blood lymphocytes (PBL) (12,000 cells/mm^3^), and/or presence of neoplastic B cells in the peripheral blood. However, according to a recent study conducted by the Ministry of Agriculture, Forestry and Fisheries of Japan, over half of EBL cattle were detected by postmortem inspections at slaughterhouses, not by on-farm inspections [2]. This indicates that the existing diagnostic criteria that rely mainly on external manifestations may be insufficient for the early detection of EBL cattle. Therefore, new diagnostic markers that can improve the detection accuracy of EBL cattle in farms are crucial. Previous studies have suggested several candidate markers whose values were associated with the development of EBL. Ishihara et al. reported significant differences in the activity values of serum total lactate dehydrogenase (LDH) and its isozymes, LDH2 and LDH3, between EBL cattle and cattle not affected by EBL (non-EBL cattle) [3]. Studies conducted by Ikeda et al. and Konnai et al. highlighted the possibility of different gene expression patterns of tumor necrosis factor receptor (TNFR) between EBL cattle and non-EBL cattle [4, 5]. Tawfeeq et al. reported the utility of serum thymidine kinase (TK) activity in the diagnosis of EBL [6]. Somura et al. reported the possibility of using the proviral load (PVL) in lymph nodes for the diagnosis of EBL [7]. However, the accuracy of these existing and candidate markers for EBL cattle detection have not been simultaneously compared using the same samples. Therefore, to evaluate the utility of these candidate markers, we investigated the discrimination capability of these markers using the blood samples of 687 cattle with different clinical states (EBL, AL, PL, and non-infected). Diagnostic accuracy was determined with a receiver operating characteristic (ROC) analysis. The influence of host factors such as sex, age, and breed were also examined to evaluate their stability as diagnostic markers.

## Results

### Test results and clinical state

**Fig. 1:**
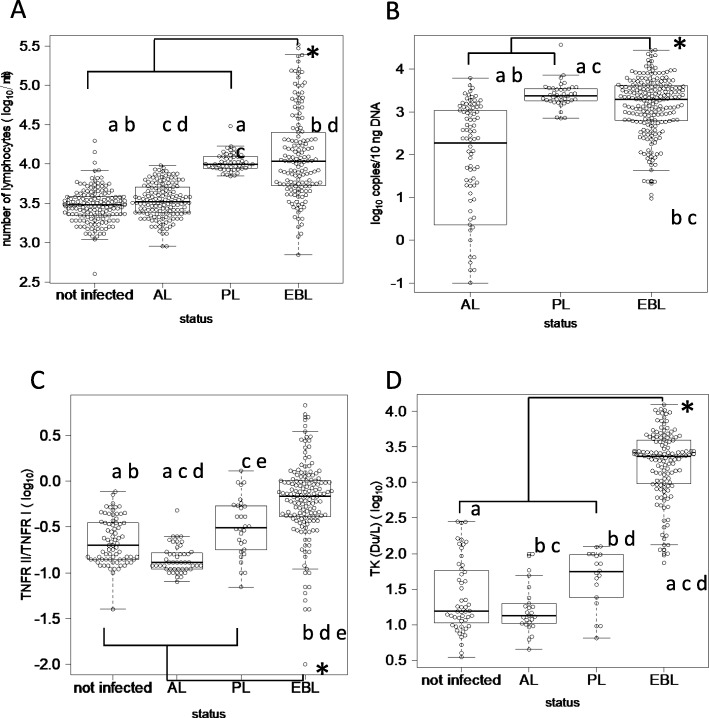
Comparison of distribution of measured values by clinical state 1 Distribution of measured values of **a** number of lymphocytes, **b** proviral load in peripheral blood, **c** TNFR II/I, and **d** TK. Each marker was compared by clinical status as follows. Non-infected: cattle not infected with bovine leukemia virus (BLV); AL: aleukemic cattle; PL: persistent lymphocytosis cattle; EBL: cattle affected with BLV-induced lymphoma. Measured values were transformed to common logarithms (log10). * indicates significant difference between EBL cattle and non-EBL cattle according to Mann-Whitney test (*p* < 0.05). Same letters indicate significant differences between BLV infection statuses as determined by the Kruskal-Wallis test and subsequent Bonferroni’s multiple comparisons test (*p* < 0.05)

**Fig. 2:**
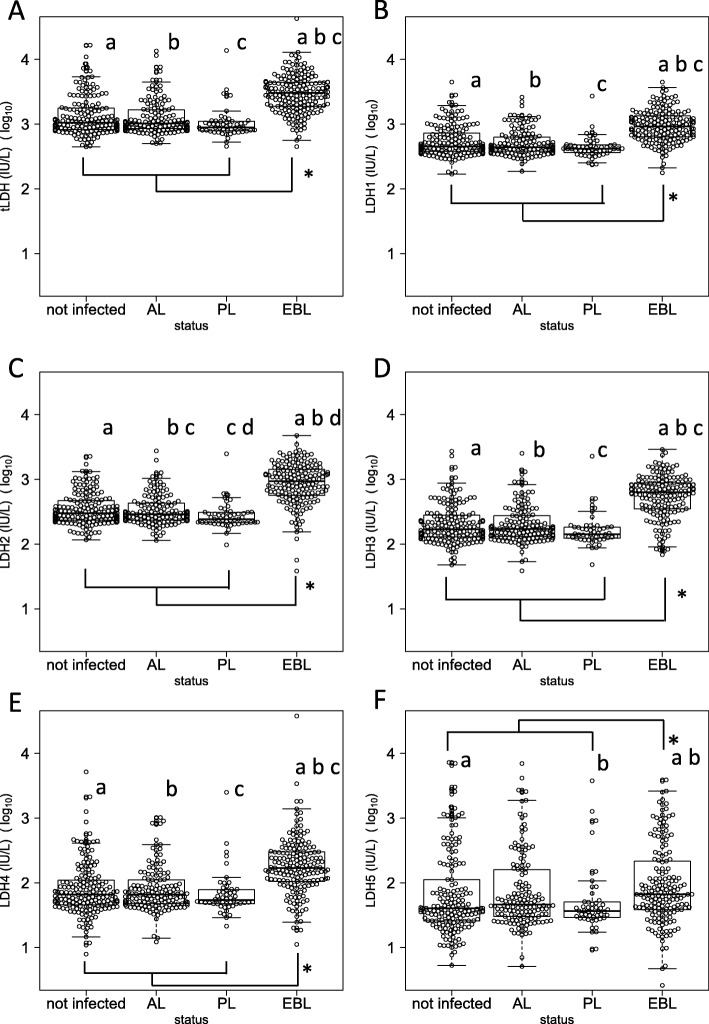
Comparison of distribution of measured values by clinical state 2 Distribution of measured values of **a** tLDH, **b** LDH1, **c** LDH2, **d** LDH3, **e** LDH4, and **f** LDH5. Each marker was compared by clinical status as follows. Non-infected: cattle not infected with bovine leukemia virus (BLV); AL: aleukemic cattle; PL: persistent lymphocytosis cattle; EBL: cattle affected with BLV-induced lymphoma. Measured values were transformed to common logarithms (log10). * indicates significant difference between EBL cattle and non-EBL cattle according to Mann-Whitney test (*p* < 0.05). Same letters indicate significant differences between BLV infection statuses as determined with the Kruskal-Wallis test and subsequent Bonferroni’s multiple comparisons test (*p* < 0.05)

The summary of values (mean, 95 percentile, and median) of each marker in EBL and no-EBL cattle were shown in Table 1. Although the values of each marker were widely distributed in both groups (EBL and non-EBL cattle), the values of all markers in EBL cattle were significantly higher than those of non-EBL cattle (*p* < 0.05 in all markers). However, when comparing values between different BLV infection status, the numbers of lymphocytes (*p* = 1.0) and PVL (*p* = 0.3) did not differ significantly between EBL and PL cattle. The values of each marker by clinical state are shown in Figs. 1 and 2. The numbers of lymphocytes, PVL, TNFRII/I, TK, and LDH4–5 for each BLV infection status were widely distributed, while the distribution of the values of total LDH and LDH1–3 were relatively narrow.

**Table 1:** Test results for each marker and the results of ROC analysis

Marker^*1^	Unit	EBL cattle^*2^		Non-EBL cattle^*3, *4^		Thresholds^*5^	AUC (95%CI)^*6^	Se (%)^*7^	Sp (%)^*8^
		Mean	Median (95 percentile)	Mean	Median (95 percentile)				
TK	Du/L	2805.6	2321 (97.2–10,031.0)	23.73	12.5 (−70.475–162.8)	99.7	0.98 (0.96–1.00)	97.1	97.0
LDH2	IU/L	1084.1	936.9 (169.5–2602.3)	397.8	288 (146.2–1244.4)	460.6	0.87 (0.83–0.91)	82.8	81.7
Total LDH	IU/L	3743	3038 (812.3–10,010.1)	1695.1	1002 (544.0–7428.8)	1389	0.85 (0.81–0.89)	87.5	75.8
LDH3	IU/L	693.9	634.4 (86.0–1735.7)	267.2	168.2 (75.2–1048.8)	283.7	0.85 (0.80–0.89)	82.2	79.7
TNFR II/I	–	0.93	0.68 (0.06–4.15)	0.26	0.17 (0.09–0.80)	0.3	0.85 (0.79–0.90)	81.7	81.8
LDH1	IU/L	1100.3	920.7 (335.6–2708.6)	602.7	438.5 (270.2–1843.5)	564.5	0.83 (0.79–0.87)	82.2	75.1
LDH4	IU/L	485.5	168.6 (24.9–1330.9)	143.6	63.9 (20.5–829.0)	91.6	0.77 (0.72–0.82)	79.3	74.6
No. of lymphocytes	102/ul	316.8	108 (16.7–2112.0)	46.4	34 (13.0–138.3)	75.5	0.76 (0.70–0.81)	61.3	73.2
PVL	copies/10 ng DNA	3289.8	1969.3 (28.3–16,224.0)	1536.6	779.9 (0.0–5729.7)	3433.1	0.69 (0.63–0.75)	51.9	72.4
LDH5	IU/L	289.94	66.45 (9.7–1881.9)	285.76	41.5 (10.5–2574.0)	54.5	0.6 (0.54–0.66)	60.9	62.9

*1 TK: thymidine kinase; LDH: lactate dehydrogenase; TNFR: tumor necrosis factor receptor; PVL: proviral load. *2 EBL cattle: cattle affected with BLV-induced lymphoma; 95 percentile: lower and upper limit of 95 percemtile *3 Non-EBL cattle: cattle without BLV-induced lymphoma (including non-infected cattle) *4 Data from non-infected cattle were not included for statistical analysis of PVL *5 Thresholds were calculated by using receiver operating characteristic (ROC) analysis *6 AUC: Area under the curve at the threshold *7 Se: sensitivity for the detection of EBL cattle at the threshold *8 Sp: specificity for the detection of EBL cattle at the threshold

### Influence of host factors

No significant differences were observed in any marker when comparing values by age. In contrast, LDH5 activity values and the number of lymphocytes were significantly different between dairy and beef cattle (*p* < 0.05). Significant differences in values between female cattle and male/castrated male cattle were observed for all markers except TK and PVL.

### Marker accuracy

ROC curve analysis results are summarized in Table 1. Values of the area under the curve (AUC), sensitivity (Se), and specificity (Sp) of TK were highest for the examined markers. Among the total LDH (tLDH) and LDH isozymes, LDH2 had the highest AUC; Se was highest in tLDH (87.5%); and Sp was highest in LDH2 (81.7%). Although the values of AUC were lower than those of LDH2, the Se (81.7%) and Sp (81.8%) of TNFR II/I were almost equivalent to those of LDH2. The Se and Sp of LDH4 and 5, number of lymphocytes, and PVL were lower than those of other markers.

## Discussion

To improve the detection accuracy of EBL cattle on farms, we evaluated the utility of several markers considered relevant to EBL development. As shown in Fig. 1, the values among EBL cattle were significantly higher than those among non-EBL cattle (i.e. PL, AL, and non-infected cattle) for all tested markers in this study.

To avoid the overestimation of Se to detect EBL cattle, we removed the data from non-infected cattle in the ROC analysis; we thereby focused on the ability of each marker to discriminate EBL cattle from PL and AL cattle. The ROC analysis using The BLV-infected samples revealed that AUC was highest in TK, followed by LDH2. TK and LDH are enzymes released into circulation from damaged or disrupted cells following neoplastic cell proliferation [8, 9]. These enzymes reflect the presence of neoplastic cells existing elsewhere in the bodies of EBL cattle and may account for the high Se of these markers. Conversely, in PL cattle, although the number of B cells is relatively high, cell proliferation is not neoplastic. Generally, normal cells seldom disintegrate during proliferation [8]; thus, the values of TK and LDH would not increase in PL cattle. Accordingly, TK and LDH demonstrated efficacy in discriminating EBL from PL cattle, resulting in high Sp of these markers for EBL cattle detection. By contrast, the number of lymphocytes and PVL only reflect the number of PBL. The low Se of the number of lymphocytes and PVL would indicate a relatively low entry rate of neoplastic cells from tumor tissue into the circulation in EBL cattle. Moreover, these two markers could not distinguish neoplastic cells from non-neoplastic cells; thus, cannot be used to discriminate EBL from PL cattle with a high number of lymphocytes and high PVL. Consequently, this would result in the low Sp of these two markers for EBL detection.

In this study, the expression levels of TNFRI and TNFRII were significantly lower in PL and EBL cattle than in AL and non-infected cattle (data not shown); these results were partially consistent with a previous study [10]. However, the ratio of the relative expression level of TNFRII to that of TNFRI (TNFR II/I ratio) was significantly higher in EBL cattle than in other cattle. Yang et al. reported that TNFRII promotes a substantial degree of cell activation, migration, and proliferation [11]. Therefore, a higher TNFR II/I ratio would indicate more neoplastic PBL, thus accounting for the higher Sp of this marker.

To further evaluate the utility of these markers to diagnosis, the influence of host factors, such as age, breed, and sex, on each marker was evaluated. TK activity value was not influenced by any of these items, while the values of tLDH, LDH 2, and 3 were significantly different between male/castrated male and female cattle. Previous studies have reported a significant difference in tLDH activity value between beef and dairy cattle [12]. In this study, 85.5% (47/55) of male/castrated cattle were beef cattle; therefore, a significant difference in the activity values of total LDH and its isozymes (LDHs) between sexes may reflect breed-specific differences. The potential influence of sex and/or breed on LDHs highlights the need for further investigation to determine threshold values for each sex or breed for LDHs.

As a diagnostic test used in the eradication of EBL cattle, high accuracy, accessibility, and simple protocol are required. The results of the ROC curve analysis in this study revealed the high accuracy of TK in detecting EBL cattle. Although the methods of measuring TK were different, these results were consistent with those of a previous study that reported the utility of TK for EBL cattle detection [6]. The enzyme-linked immunosorbent assay (ELISA) kit used for measuring TK activity values in this study is accessible, easy to use, and is thus suitable for diagnostic purposes. Similarly, the kits for measuring activity values of LDHs are also accessible, and the test methods are routinely conducted in veterinary practice [13–15]. Thus, measuring the active values of LDHs would also be suitable for diagnostic purposes. Nevertheless, these two enzymes are not specific markers for EBL cattle. The activity values of these enzymes also reflect cancers other than EBL and inflammation. Tawfeeq et al. investigated TK activity values in cattle with tumors other than EBL and inflammatory diseases and showed that 15.3% (2/13) of cattle with other tumors and 21.4% (4/14) of cattle with inflammatory diseases had higher activity values than their cut-off point [6]. Our preliminary investigations using 73 cattle affected by diseases other than EBL (pericarditis, pneumonia, mastitis, milk fever, abomasal displacement, hepatitis, arthritis, etc.) revealed that 30.1% (22 /73) of these cattle had higher tLDH activity values than the threshold value (1398.0 IU/L) determined in this study. The increases in TK and tLDH values caused by these diseases suggest that the cattle infected with other viruses that induce inflammation and necrosis would also be associated with higher values of these markers. Therefore, further evaluation of the influence of viral infections or other health disorders on the values of TK and LDHs is warranted. However, TK and LDHs are at present acceptable for screening EBL cattle when positive cattle are confirmed by other tests, such as rectal palpation and blood smear examination. The application of TK and/or LDH values for screening prior to the performance of diagnostic tests would improve the cost effectiveness of detecting EBL cattle on farms without compromising accuracy and would thus contribute to the reduction of economic loss of farms.

## Conclusions

The accuracy of several diagnostic markers detectable in the blood were compared in this study to evaluate their utility in detecting EBL cattle on farms. Statistical analyses revealed that TK and LDH2 had high Se and Sp, and these serum enzymes could discriminate EBL cattle from PL cattle. The measurement methods for these enzymes have high accuracy and are accessible and easy. Therefore, TK and LDHs could help to screen EBL cattle antemortem. Although caution should be exercised when interpreting sexual differences in LDHs, the combined use of these enzymes and existing diagnostic tests would improve the efficiency of detecting EBL cattle on farms undergoing EBL eradication programs.

## Methods

### Samples

EDTA-treated whole blood or PBL, sera, and information (age, sex, breed, clinical findings, and diagnosis on farms) of 687 tested cattle collected between 2010 and 2015 were provided by 26 animal hygiene service centers in 17 prefectures and six meat inspection centers in six prefectures. The cattle met the following classification criteria based on inspections conducted in animal hygiene service centers or meat inspection centers by the official veterinarians of each facility. “EBL cattle”: tumors were observed with neoplastic B cells and classified as positive based on anti-BLV antibody detection using a commercial ELISA kit (JNC, Tokyo, Japan). Cattle that did not meet these criteria or were alive for more than 1 year after sample collection were classified as “non-EBL cattle”. Among non-EBL cattle, those that were negative for both BLV antibody test using the ELISA kit and BLV proviral DNA detection by real time PCR were classified as “non-infected cattle”. Non-EBL cattle that were positive for either of these tests were classified as “BLV-infected cattle”. BLV-infected cattle were classified as “PL (persistent lymphocytosis) cattle” if they met the criteria of Bendixen’s key [16]. BLV-infected cattle that did not fulfill Bendixen’s key were classified as “AL (aleukemic) cattle.” The populations and details of the cattle in each group are presented in Table 2. Some samples were not tested for any of the investigated markers due to the shortage of sample volume or insufficient sample conditions. The numbers of samples tested for each marker is presented in Table 3.

**Table 2:** Details of cattle used in this study

Status^*1^		Total	Age (years old)				Sex			Breed		
			2>	3–5	6	UK^*2^	Female	Male/ Castrated	UK	Dairy	Beef	UK^*2^
EBL		281	48	97	128	8	253	23	5	166	113	2
Non_EBL	Non-infected	208	109	46	47	6	174	28	6	120	85	3
	AL	147	33	58	56	0	143	4	0	92	55	0
	PL	51	3	23	25	0	51	0	0	49	2	0
Total		687	193	224	256	14	621	55	11	427	255	5

*1 EBL: cattle with BLV-induced lymphoma; non-infected: cattle without BLV infection; AL: aleukemic cattle; PL: persistent lymphocytosis cattle; *2 UK: unknown

**Table 3:** Number of tested cattle for each marker

Number of cattle	Status*			
	EBL	Non-infected	AL	PL
Total	281	208	147	51
Number of lymphocytes	150	160	147	51
PVL	210	149	94	40
tLDH	184	178	147	51
LDH isozyme	174	174	146	51
TK	138	65	44	22
TNFR II/I	153	76	47	30

*EBL: cattle with BLV-induced lymphoma; non-infected: cattle without BLV infection; AL: aleukemic cattle; PL: persistent lymphocytosis cattle

### Leukocyte count

The numbers of leukocytes and lymphocytes in EDTA-treated whole blood were quantified using a veterinary hematology analyzer (Celltac Alpha, MEK-6450; Nihon-Kohden, Tokyo, Japan).

### PBL isolation

PBL was isolated from EDTA-treated whole blood according to the method previously described by Asfaw et al. [17]. Isolated PBL was divided equally for DNA and RNA extraction. The PBL for RNA extraction was suspended in 1 mL of TRI Reagent (Molecular Research Center, Inc., OH, USA). All samples were stored at − 20 °C for further experiments.

### Quantification of BLV PVL

DNA was extracted from PBL using a commercial DNA extraction kit (DNeasy Blood & Tissue Kit; Qiagen, Hilden, Germany) according to the manufacturer’s instructions. To detect and quantify BLV proviral DNA, real-time PCR using a TaqMan probe was performed as described previously [18].

### Quantification of bovine tumor necrosis factor receptor mRNAs

Total RNA was extracted from PBL suspended by TRI Reagent according to the manufacturer’s instructions. To remove the residual DNA, the extracted RNA was treated with DNase I (Qiagen) for 15 min at room temperature on the column membrane attached to the RNeasy Mini Kit (Qiagen) according to the manufacturer’s instructions. As a positive control for real time PCR, synthetic RNA of bovine TNFRI (209 base), TNFRII (255 base), and β-actin (251 base) genes were produced from total RNA extracted from PBL of non-infected cattle by the following process. First, reverse transcriptase (RT)-PCR targeting TNFRI, TNFRII, and β-actin was performed with the primers listed in Table 4. Primers of each gene were designed based on the primer sequence described in previous studies [5, 19]. RT-PCR was performed using the Gene Amp PCR System 9700 (Thermo Fisher Scientific, Waltham, MA, USA) with PrimeScript One Step RT-PCR Kit Ver.2 kit (Takara Bio Inc., Shiga, Japan) according to the manufacturer’s instructions at a final reaction volume of 25 μL. Each reaction mixture contained 0.4 μM of each primer and 2 μL of RNA sample. RT-PCR was performed as follows: RT reaction at 50 °C for 30 min and initial denaturation at 94 °C for 2 min, followed by 30 cycles of denaturation at 94 °C for 20 s, annealing at 55 °C for 20 s, and extension at 72 °C for 20 s. Following sequence confirmation, each purified PCR product was used as a linear template DNA for in vitro transcription reaction. The in vitro transcription reaction was conducted with the in vitro Transcription T7 Kit (Takara Bio Inc.) according to the manufacturer’s instructions. Transcript RNA was purified using the same protocol with removal of residual DNA from RNA samples. Copy number of each transcript RNA was calculated from the concentration and molecular weight of each transcript RNA. Transcript RNA was serially diluted ranging from 5 × 10^2^ to 5 × 10^8^ copies/2 μL. RT reaction was performed with 2 μL of these dilutions using Random 6mers in PrimeScript RT reagent kit (Perfect Real Time), (Takara Bio Inc.) according to the manufacturer’s instructions at a final reaction volume of 10 μL. The 2 μL of cDNA of each gene transcribed from RNA ranging from 10^2^ to 10^8^ copies was used to draw a standard quantification curve. The concentration of RNA in each sample was measured using NanoDrop ND-1000 (Thermo Fisher Scientific), and 20 ng of RNA was used for RT reaction by the same protocol with positive controls. Real time PCR was carried out using the Applied Biosystems 7500 Real-Time PCR Systems (Thermo Fisher Scientific) with SYBR Premix Ex Taq II (Tli RNaseH Plus, Takara Bio Inc) according to the manufacturer’s instructions at a final reaction volume of 25 μL. Each reaction mixture contained 0.2 μM of each primer and 2 μL of cDNA sample. Primers for TNFR I, TNFR II, and β-actin for real time PCR are described elsewhere [5, 19]. Amplification was performed according to the following conditions: initial denaturation at 95 °C for 30 s, followed by 42 cycles of denaturation at 95 °C for 5 s and annealing at 60 °C for 34 s. The dissociation stage was added after the amplification. The amplicon specificity was confirmed by melting curve analysis. The results are shown as the ratios obtained by dividing the copy numbers of TNFRI and TNFRII by that of β-actin. The difference in gene expression between TNFRI and TNFRII are shown as the ratios obtained by dividing the relative expression level of TNFRII by that of TNFRI (TNFR II/I).

**Table 4:** Primers used for RT-PCR

Gene	Primer sequence (forward and reverse)^*^	Reference No.
Bovine TNFRI	5′-GGATCCCTAATACGACTCACTATAGGGAGACGCCTCTGTCGTCTTAGCAT-3’	[5]
	5′-AAGTTCCAGT CCTGTCTCCA-3’	
Bovine TNFRII	5′-CTAATACGACTCACTATAGGGAGACTCGACCAGCAGCACGGACA-3’	
	5′-GCGTCTGTGTCCCTCGTGGA-3’	
Bovine β-actin	5′-CTAATACGACTCACTATAGGGAGACGCACCACTGGCATTGTCAT-3’	[19]
	5′-TCCAAGGCGACGTAGCAGAG-3’	

^*^T7 promoter sequence and extra nucleotides were added at 5’end of each forward primer

### Measurement of tLDH activity value

Activity value of serum tLDH was measured using the automated biochemical analyzer SPOTCHEM EZ SP-4430 (ARKRAY, Inc., Kyoto, Japan) and reagent strips exclusive for the analyzer SPOTCHEM II LDH (ARKRAY) according to the manufacturer’s instruction. The samples with tLDH activity values higher than the measurement limit of the analyzer (2000 IU/L) were adequately diluted with saline. The activity values of these samples were obtained by multiplying dilution rate by measured tLDH activity value.

### Measurement of activity value of LDH isozymes

Separation of serum LDH isozymes was performed by electrophoresis using Quickgel LD (Helena Laboratories Japan Co, Saitama, Japan) and an exclusive electrophoresis chamber (Helena Laboratories Japan Co) according to the manufacturer’s instructions. To obtain a sharply defined electrophoretic profile, samples with tLDH activity values higher than 1000 IU/L were diluted with saline to below 1000 IU/L. LDH zymograms were stained with TitanGel S-LD reagent (QG) (Helena Laboratories Japan Co) according to the manufacturer’s instructions. Stained zymograms were scanned, and the fraction rate was quantified using Jokoh densitron CR-20 (JOKOH Co., LTD. Kikukawa, Shizuoka, Japan) and QuickScan (Helena Laboratories Japan Co). The high correlativity of measurement values between CR-20 and QuickScan has been confirmed by the measurement of 67 samples (data not shown). Thus, we pooled the measurement values obtained from both densitometers for statistical analysis in this study. Each isozyme activity value was obtained by multiplying the fraction rate of each isozyme by tLDH activity value.

### Measurement of TK activity value

TK activity value in sera/plasma was measured using a commercial ELISA kit (DiviTum V2, BIOVICA International AB, Uppsala, Sweden) according to the manufacturer’s instructions. TK activity was originally determined as a ratio to the activity of a reference sample of recombinant TK in serum and expressed as DiviTum Units per liter (Du/L). According to the manufacturer’s instructions, 1000 Du/L corresponds to the activity that was obtained with 1000 ng TK/L.

### Statistical analysis

To evaluate the utility of each marker to detect EBL cattle, the values of each marker were compared between EBL cattle and non-EBL cattle and tested using the Mann–Whitney (M-W) test. To evaluate the influence of BLV infection status (AL, PL, EBL, and non-infected) on the values of these markers, the values of each marker were compared between EBL, AL, PL, and non-infected cattle and tested using Kruskal–Wallis (K-W) test followed by Bonferroni’s multiple comparisons test. To test the influence of host factors on the values of these markers, the influence of age (≤2 years, 3–5 years, and ≥ 6 years), breed (dairy cattle and beef cattle), and sex (female and male/castrated male) of tested cattle on the values of each marker was evaluated using either the M–W test or K–W test followed by Bonferroni’s multiple comparisons test. Since non-infected cattle had no proviral DNA, they were excluded from statistical comparisons of PVL. The accuracy of each marker to detect EBL cattle was validated by ROC curve analysis using Se and Sp to discriminate EBL cattle from EBL, PL, and AL cattle. In ROC curve analyses, the value of AUC was used to compare accuracy among the markers. The cut-off value that showed the highest sum of Se and Sp was considered the most favorable threshold value for the diagnosis of EBL. In all statistical tests, *p* values < 0.05 were considered significant. All statistical analyses were performed using R version 3.4.3 [20].

## Data Availability

All data included in this study are available upon request to the corresponding author.

## Abbreviations

AL: Aleukemic
AUC: Area under the curve
BLV: Bovine leukemia virus
EBL: Enzootic bovine leukosis
ELISA: Enzyme-linked immunosorbent assay
K-W: Kruskal–Wallis
LDH: Lactate dehydrogenase
LDHs: tLDH and its isozymes
M-W: Mann–Whitney
PBL: Peripheral blood leukocytes
PL: Persistent lymphocytosis
PVL: Proviral load
ROC: Receiver operating characteristic
RT: Reverse transcriptase
Se: Sensitivity
Sp: Specificity
TK: Thymidine kinase
tLDH: Total LDH
TNFR: Tumor necrosis factor receptor
